# Developing and Assessing a Scalable Digital Health Tool for Pretest Genetic Education in Patients With Early-Onset Colorectal Cancer: Mixed Methods Design

**DOI:** 10.2196/59464

**Published:** 2025-01-17

**Authors:** Jessica N Rivera Rivera, Moran Snir, Emilie Simmons, Tara Schmidlen, Misha Sholeh, Melinda Leigh Maconi, Carley Geiss, Hayden Fulton, Laura Barton, Brian D Gonzalez, Jennifer Permuth, Susan Vadaparampil

**Affiliations:** 1 Healthcare Delivery Research Network MedStar Health Research Institute Washington, DC United States; 2 Nest Genomics New York, NY United States; 3 Non-Therapeutic Research Office Moffitt Cancer Center Tampa, FL United States; 4 Participant Research, Interventions, and Measurement Core Moffitt Cancer Center Tampa, FL United States; 5 Department of Pathology Moffitt Cancer Center Tampa, FL United States; 6 Research Diversity and Workforce Development Moffitt Cancer Center Tampa, FL United States; 7 Department of Gastrointestinal Oncology Moffitt Cancer Center Tampa, FL United States; 8 Department of Health Outcomes and Behavior Moffitt Cancer Center Tampa, FL United States

**Keywords:** genetic education, genetic testing, genetic counseling, digital health, early-onset colorectal cancer

## Abstract

**Background:**

National guidelines recommend germline genetic testing (GT) for all patients with early-onset colorectal cancer. With recent advances in targeted therapies and GT, these guidelines are expected to expand to include broader groups of patients with colorectal cancer. However, there is a shortage of genetic professionals to provide the necessary education and support for informed consent. As such, there is a pressing need to identify alternative approaches to facilitate and expedite access to GT.

**Objective:**

This study describes the development of a pretest education intervention, Nest-CRC, to facilitate the uptake of germline GT among patients with early-onset colorectal cancer. Patients with early-onset colorectal cancer and health care providers reviewed Nest-CRC, and their reactions and recommendations were captured using a nested mixed methods approach.

**Methods:**

Using the learner verification approach, we conducted 2 sequential phases of surveys and interviews with English- and Spanish-speaking patients with early-onset colorectal cancer and health care providers. The surveys assessed participants’ experiences with genetic services and provided immediate feedback on the Nest-CRC genetic education modules. Semistructured interviews evaluated participants’ perceptions of self-efficacy, attraction, comprehension, cultural acceptability, and usability of Nest-CRC. Survey data were analyzed using descriptive statistics (mean, median, and proportions), while interview data were analyzed through line-by-line coding of the transcribed interviews. After each phase, Nest-CRC was refined based on participants’ recommendations.

**Results:**

A total of 52 participants, including 39 patients with early-onset colorectal cancer and 13 providers, participated in the study. Of these, 19 patients and 6 providers participated in phase 1 (N=25), and 20 patients and 7 providers participated in phase 2 (N=27). Most participants (phase 1: 23/25, 92%, to 25/25, 100%; phase 2: 24/27, 89%, to 27/27, 100%) agreed that each of the 5 education modules was easy to understand and helpful; 13 patients reported no history of GT, with 11 (85%) expressing interest in GT and 2 (15%) remaining unsure after completing Nest-CRC. Participants reported that Nest-CRC provided sufficient information to help them decide about GT. The tool was deemed acceptable by individuals from diverse backgrounds, and participants found it visually attractive, easy to comprehend, and user-friendly.

**Conclusions:**

The findings revealed that Nest-CRC is a promising strategy for facilitating pretest education and promoting GT. Nest-CRC has been refined based on participant recommendations and will be re-evaluated.

## Introduction

Colorectal cancer (CRC) is the third most common cause of cancer incidence and mortality among men and women in the United States [1]. By 2030, it is projected to become the leading cause of death among patients diagnosed with early-onset cancer (under the age of 50 years) [2]. Approximately 14%-25% of early-onset CRCs are linked to hereditary factors, irrespective of family history [3,4]. Identifying germline variants in patients with CRC can help reduce morbidity and mortality by enabling guided treatment decisions, risk management to prevent and detect new primary cancers early, and cascade testing for at-risk relatives [5-9]. The National Comprehensive Cancer Network (NCCN) Guidelines recommend multigene panel testing (MGPT) for *all* individuals diagnosed with CRC before the age of 50 years and consider its use for all individuals diagnosed with CRC [10]. MGPT is recommended because it can simultaneously identify gene variants associated with various cancers and simplifies referrals for genetic testing (GT), as neither family history nor patient tumor characteristics are required.

Genetic services heavily depend on clinicians to identify and refer high-risk patients for genetic counseling (GC) and GT. However, approximately 40% of patients with early-onset CRC are not referred for GC [11,12]. Additionally, racial and ethnic disparities exist in germline studies and access to genetic services [11,13]. A study conducted between 2009 and 2017, involving patients with early-onset CRC treated at a tertiary-care referral center and a safety-net health system, found that Black patients were less likely to attend GC compared with Hispanic and non–Hispanic White patients [11]. Another retrospective study, using data from 2012 to 2016 across 4 academic medical centers, found that Black and Hispanic patients with CRC were referred to genetic specialists less often than non–Hispanic White patients [13]. However, among those referred, no racial or ethnic differences were observed in GC attendance [13], highlighting a missed opportunity for guideline-concordant genetic care. Therefore, systematic strategies are necessary to ensure GT services are offered to all patients with early-onset CRC.

With the expanding indications for genetic services, there is a shortage of genetic counselors and qualified genetic professionals [14,15], which can result in delays in GT. Although oncologists and other health care providers can order GT for at-risk patients, most lack the expertise or time to provide adequate genetic education [16,17]. Consequently, the American Society of Clinical Oncology (ASCO) acknowledges GC as the standard of care but also advocates for alternative approaches to delivering genetic services [18]. Studies examining the uptake of genetic services among patients with early-onset CRC have identified cost, limited availability of services, and racial and ethnic referral disparities as key barriers [11,19]. To address these known barriers to GT, the National Institute of Health Clinical Genome Resource’s Consent and Disclosure Recommendations Working Group suggested reserving traditional, provider-delivered pre- and posttest GC for patients with greater clinical and genetic complexity, such as those with conditions lacking well-established testing and risk management criteria [20]. Alternative approaches to genetic education are needed to facilitate, expedite, and expand access to GT for patients at risk of cancer without overburdening GC resources [21]. To address this, we developed a digital health tool designed for patients with early-onset CRC from diverse racial and ethnic backgrounds. This tool systematically delivers pretest education and triages patients to GC and GT.

Previous studies involving patients with cancer suggest that digital genetic education is well-accepted and effective in improving knowledge, decisional satisfaction, and reducing decisional conflict [22-24]. However, most educational interventions addressing germline testing have focused on patients with breast cancer [25-27]. To our knowledge, only 2 studies have evaluated alternative strategies for genetic education in patients with CRC. These studies were not specific to patients with early-onset CRC, and their educational content focused on tumor testing [28] or GC [29]. Therefore, in this study, we propose a digital health tool designed for patients with early-onset CRC to promote autonomy by allowing them to access relevant germline information at their convenience, make informed decisions about GT, and opt-in to pretest GC if desired. This study outlines patient and provider feedback on the digital pretest genetic education tool and provides recommendations for its implementation, using a mixed methods approach.

## Methods

### Intervention

**Figure 1:**
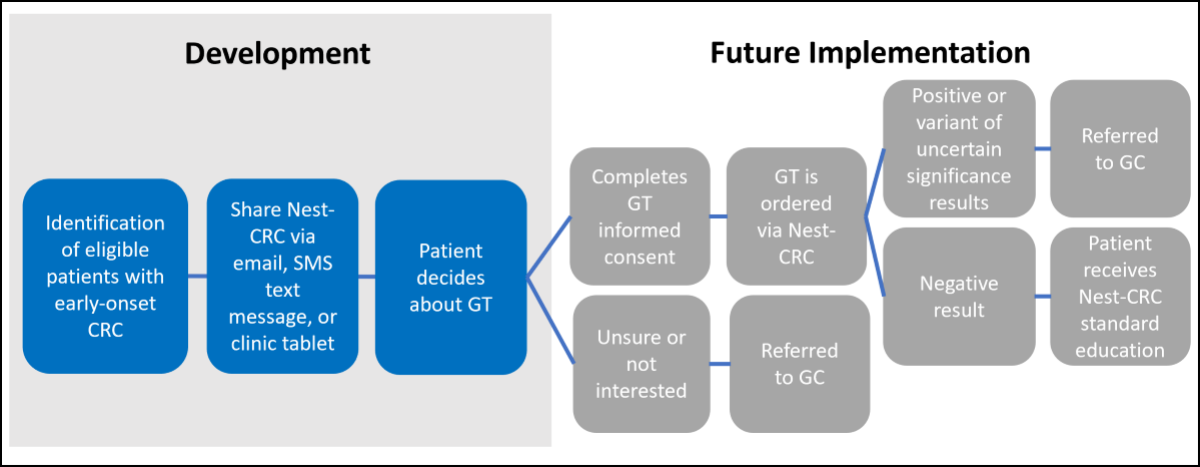
Development and future implementation of the Nest-CRC tool. CRC: colorectal cancer; GC: genetic counseling; GT: genetic testing.

Nest Genomics is a software company specializing in developing tools that help patients and providers scale the delivery and long-term implementation of genomic information. The Nest platform is a comprehensive, Health Insurance Portability and Accountability Act (HIPAA)–compliant solution designed to launch, implement, and scale longitudinal genomic programs, supporting both patients and clinicians throughout the care continuum—from patient identification and education to test ordering, result integration, and long-term management. Within Nest, our research team developed the Nest-CRC, a digital health tool designed to provide pretest genetic education for patients with early-onset CRC from diverse racial and ethnic groups (Figure 1). Nest-CRC is not publicly available at this time. The tool is divided into 5 brief modules. The modules included in the first version of Nest-CRC are (1) hereditary CRC, (2) GT, (3) benefits and risks, (4) care recommendations, and (5) implications for family members. Nest-CRC takes approximately 10 minutes to complete. The information covered in Nest-CRC is supported by ASCO content recommendations for pretest genetic education [18], standard informed consent for GT, and feedback from genetic counselors and experts on the study team. Nest-CRC delivers education through text and images and is accessible on any personal device with internet access [30]. It includes images that are representative of different ages and races, with written content at a 5th-grade reading level in both English and Spanish.

### Procedure

We conducted a nested mixed methods study to develop and refine Nest-CRC for patients with early-onset CRC. Following the learner verification approach [31], we carried out 2 sequential phases of patient and provider surveys and interviews about Nest-CRC. Learner verification, which is useful for formative research, uses semistructured individual interviews to assess the appropriateness of materials for a target population. The quantitative data collected in each phase were used to describe the demographic characteristics of our sample population, their experiences with genetic services, and to obtain immediate feedback on their experience navigating each of the genetic education modules.

**Figure 2:**
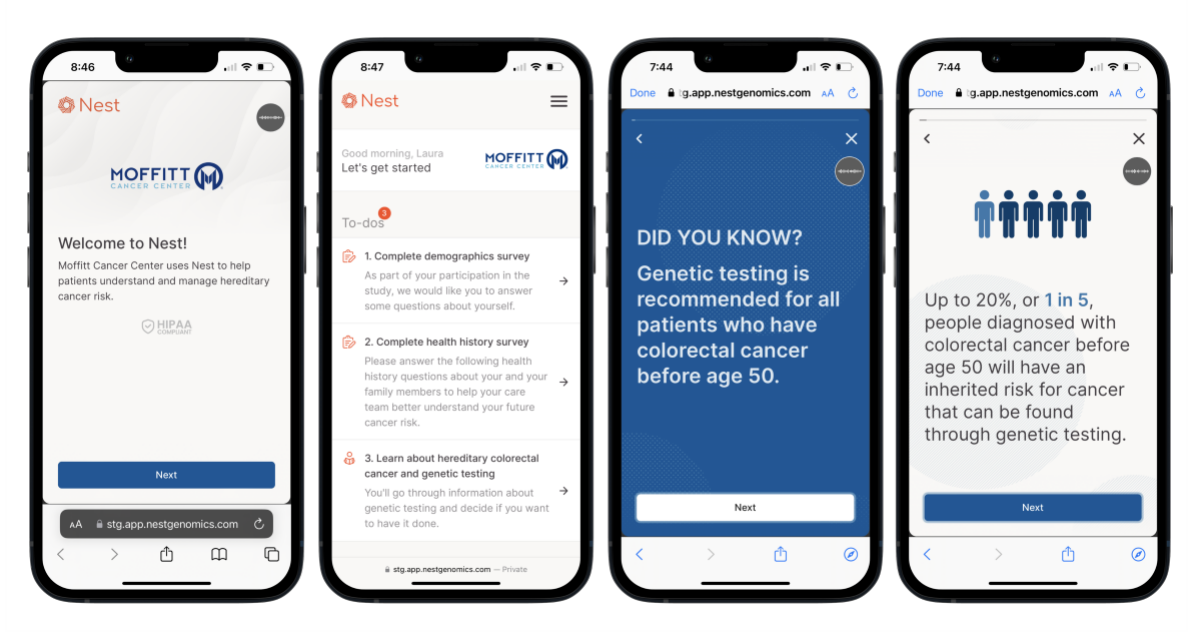
Example of Nest-CRC for phase 2.

During phase 1, participants were emailed a link to a brief survey covering demographics, clinical characteristics, and experiences with genetic services, as well as the Nest-CRC educational modules, which included integrated questions about each module. Participants completed a semistructured interview after finishing the survey and Nest-CRC education. Patients could complete the survey and Nest-CRC on their personal device or a clinic tablet. After phase 1, we refined Nest-CRC based on participant recommendations (Figure 2). The revised version of Nest-CRC was then re-evaluated in phase 2 using the same procedure as in phase 1. However, in phase 2, participants also had the option to receive the link via SMS text message. Each version of Nest-CRC was reviewed by the entire study team for final edits.

### Ethical Considerations

This study was reviewed and approved by the Moffitt Cancer Center’s Scientific Research Committee and Institutional Review Board (approval number 22176) on November 15, 2022. Before enrollment, all participants were provided with a copy of the informed consent form. All patients gave verbal informed consent in person or over the phone, and all providers consented to participate via email. Participants who agreed to take part in the study received a link to the survey and Nest-CRC education. The interviewer reviewed the informed consent form again with each participant before beginning the interview. All data collected for this study were deidentified using a unique ID number. Participants received US $25 upon completion of the interview.

### Recruitment

From February to August 2023, English- and Spanish-speaking adult patients with early-onset CRC with upcoming medical appointments at the Moffitt Cancer Center Gastroenterology clinic were contacted in the clinic or by phone and invited to participate in the study. Recruitment flyers in both English and Spanish were posted in various waiting areas at Moffitt Cancer Center and distributed to community partners for sharing on their social media platforms (eg, Facebook). Interested potential participants responded to flyers and internet advertisements by calling or emailing the study team. The study team then contacted these individuals to screen them, obtain informed consent, and schedule their interview.

We purposely recruited at least 20% (n=4) Spanish-speaking and 20% (n=4) Black patients for each phase. These groups were specifically targeted because a lower proportion of Spanish-speaking and Black patients seen at the oncology clinic were eligible for the study. Gastroenterologists, oncologists, nurse practitioners, and genetic counselors with at least 2 years of experience working with patients with CRC were recruited from Moffitt Cancer Center (phases 1 and 2), MedStar Health (phase 2 only), and through referrals (phases 1 and 2). Different individuals participated in each phase.

### Survey Measures

Before reviewing Nest-CRC, participants were asked to complete a brief survey capturing relevant sociodemographic, clinical, and epidemiologic characteristics adapted from the Health Information National Trends Survey (HINTS) 5 [32]. Patients were asked about their age, gender, race, ethnicity, country of birth, marital status, education level, employment status, household income, and insurance, while providers were asked about their age, gender, marital status, race, ethnicity, and professional degree. Additional information collected from patients included self-reported technology literacy (using the 3-item Digital Health Care Literacy Scale [33]), health literacy (using the 3-item Short Literacy Survey [34]), clinical details about their cancer diagnosis (including the type of CRC, cancer stage, age of diagnosis, and treatment history), family history of early-onset CRC, Ashkenazi Jewish ancestry (due to the known genetic risk for CRC and other cancers in this population), awareness of genetic services (ie, GC, GT, hereditary cancers, and Lynch syndrome), and history of genetic services (ie, referrals to genetic services, GC, and GT). We also evaluated patients’ perceived importance of GT for cancer prevention and early detection (adapted from HINTS 5) [32]. Providers’ self-reported practice characteristics included the frequency of communication with patients about genetic risk, referrals to GT, working with patients with early-onset CRC, and working with ethnic/racial minority patients. At the end of each module (n=5), participants were asked 2 questions: whether the information was easy to understand and whether it was helpful, using a 5-point Likert scale ranging from 1=strongly disagree to 5=strongly agree. Responses of strongly agree and agree were recoded as agreed, while neither agree nor disagree, disagree, and strongly disagree were recoded as did not agree. Upon completing Nest-CRC, participants were asked if they were interested in GT, with response options of yes, no, or unsure.

### Interview Process

Interview guides were based on key elements of learner verification. Patients and providers gave feedback on the Nest-CRC tool’s attractiveness, comprehension, self-efficacy, cultural acceptability, and usability (Table 1). The development of these questions was informed by prior studies using the learner verification approach [31,35,36] and refined by the study team. All interviews were recorded, and audio files were transcribed verbatim. Interviews conducted in Spanish were translated into English [37]. JRR conducted all English and Spanish interviews via Zoom (Zoom Communications/Qumu Corporation; phase 1: mean 26.96 minutes, range 17.26-37.36 minutes; phase 2: mean 26.04 minutes, range 15.07-36.02 minutes).

**Table 1:** Sample questions included in the interviews.

Key elements	Example of questions
Attractiveness	What was the first thing that came to your mind when using the Nest-CRC tool? How do you feel after going through this tool? What attracted you or did not attract you about Nest-CRC?
Comprehension	Overall, did you find the tool easy to understand?/Overall, did you find the tool easy to understand for patients that you typically see in the clinic?^a^ While completing Nest-CRC, can you describe any words, phrases, or sections that were difficult to understand? What information do you think might be missing from the genetic education?
Self-efficacy	Do you think this tool provides enough information to make an informed decision about getting or not genetic testing? After completing the Nest-CRC tool, can you give me some examples of what happens after genetic testing?/After completing the Nest-CRC tool, would the patients have enough information for getting genetic testing?^a^
Cultural acceptability	What are your thoughts about Nest-CRC being appealing to people from different backgrounds? Were there any sections of the genetic education that made you feel uncomfortable?/Were there any sections of the genetic education that made you feel uncomfortable, or do you think patients might feel uncomfortable?^a^
Usability	Overall, did you have any challenges using the tool? Would you recommend this tool to other patients with colorectal cancer?/Would you recommend this tool to patients with early-onset colorectal cancer?^a^ If your health care provider had referred you to this tool, would you have completed it?/Would you think your patients will complete this tool?^a^ What do you think is the best way to share this tool with other patients with colorectal cancer diagnosed before the age of 50 years? How should this tool be used in the clinic?^a^

^a^Provider-specific questions.

### Data Analysis

Descriptive statistics (mean, median, and proportions) were calculated using IBM SPSS software (version 28). All qualitative data were transcribed into English and reviewed by team members. The research team met to develop the initial codebook, using deductive codes derived from the key elements of the interview guides. The themes and codebook, along with operational definitions for each code, were subsequently refined during the intercoder reliability process. Three research team members (CG, MLM, and HF) coded the transcripts using a direct content analysis approach. Intercoder reliability was assessed until Cohen κ reached 0.80, indicating substantial agreement [38]. Qualitative analysts performed line-by-line coding of all interview data using NVivo 12 software (Lumivero).

## Results

### Demographics and Clinical Characteristics

#### Phase 1

**Figure 3:**
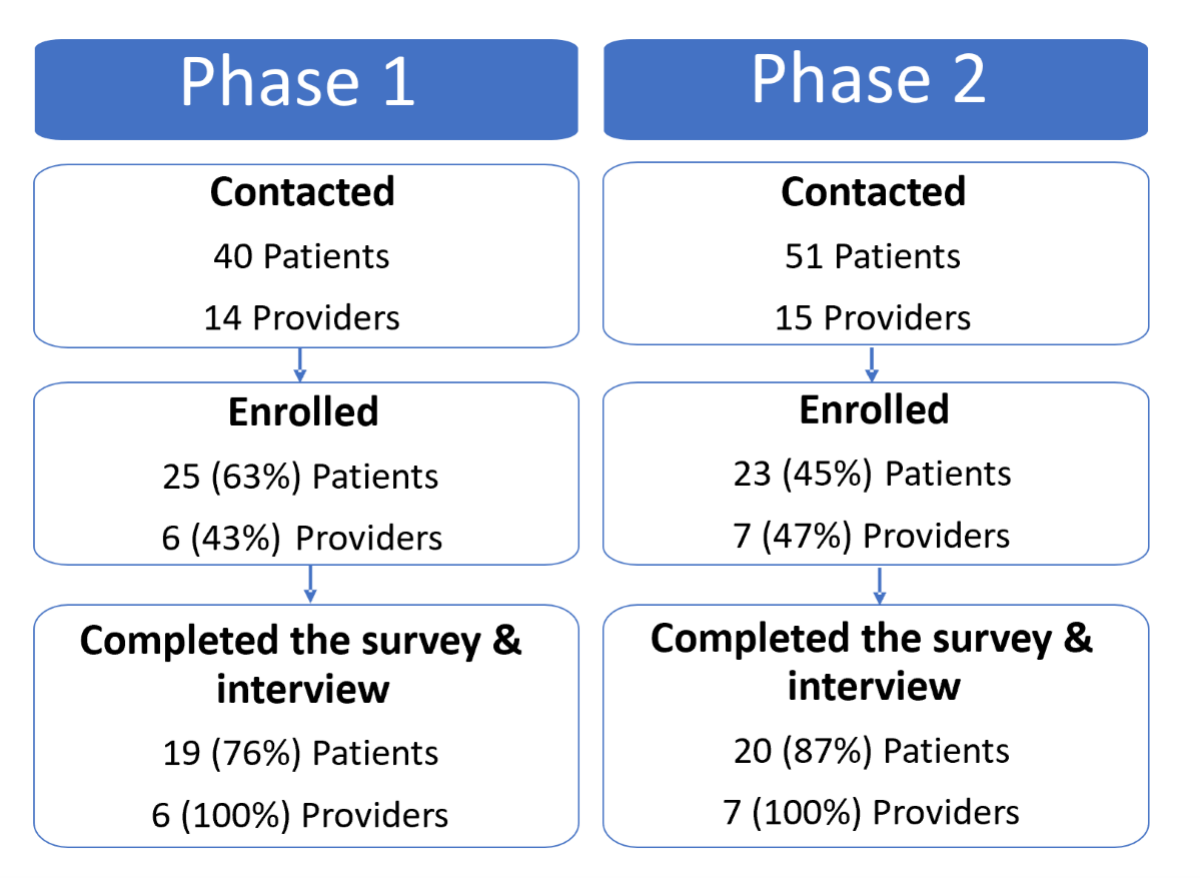
Recruitment study flow for phases 1 and 2.

We contacted a total of 40 patients with early-onset CRC and 14 providers, of whom 19 patients and 6 providers completed the survey and interview (Figure 3). The most common reasons patients did not participate were a lack of interest or unsuitable timing due to their recent diagnosis and treatment. Providers generally declined participation passively.

The median age of patients was 43 (range 27-51) years (Tables 2 and 3), with 10 out of 19 (53%) being female, 2 (11%) identifying as Black, 6 (32%) of Hispanic ethnicity, and 4 (21%) preferring Spanish (Table 1). About half (n=10) had at least some college education, and most were employed (n=11). One-third of the patients had stage 4 cancer (n=7), and half were undergoing active treatment (n=9). Most patients reported adequate health literacy (median 4, range 1-4) and technology literacy (median 4, range 1.6-4). Before completing Nest-CRC, 18 of the 19 (95%) patients indicated that GT was very important for cancer prevention and early detection, though only 10 (53%) and 15 (79%) reported awareness of GC and GT, respectively.

The median age of providers was 39 (range 31-46) years (Table 4). Half of the providers were female, all identified as White, and 1 was Hispanic and preferred Spanish. Two-thirds were medical or surgical oncologists, and 2 were genetic counselors. All providers had at least 2 years of experience with patients with CRC (median 11 years, range 2-13) and discussed genetic risk with patients at least 50% of the time.

**Table 2:** Patients’ demographic characteristics by phase.

Demographic			Phase 1 (n=19)		Phase 2 (n=20)
Age (years), median (range)			43 (27-51)		47 (36-59)
**Gender, n (%)**					
	Female	10 (53)		11 (55)	
	Male	9 (47)		9 (45)	
**Race, n (%)**					
	White only	14 (74)		12 (60)	
	Black only	2 (11)		4 (20)	
	More than one race	2 (11)		3 (15)	
	Other	1 (5)		1 (5)	
Hispanic ethnicity, n (%)			6 (32)		7 (35)
Spanish-preferring, n (%)			4 (21)		4 (20)
Born in the US mainland, n (%)			15 (79)		14 (70)
**Marital status, n (%)**					
	Married/partnered	13 (68)		14 (70)	
	Single	4 (21)		2 (10)	
	Divorced/separated	2 (11)		4 (20)	
**Education, n (%)**					
	<High school diploma	2 (11)		0 (0)	
	High school diploma or General Educational Development	6 (32)		1 (5)	
	Some college/vocational school	4 (21)		6 (30)	
	≥College graduate	7 (37)		13 (65)	
**Employment status, n (%)**					
	Employed^a^	12 (63)		14 (70)	
	Unemployed	3 (16)		2 (10)	
	Homemaker	1 (5)		1 (5)	
	Disable	3 (16)		3 (15)	
**Annual household income (US $), n (%)**					
	<19,999	3 (16)		3 (15)	
	20,000-49,999	5 (26)		0 (0)	
	50,000-99,999	2 (11)		4 (20)	
	≥100,000	8 (42)		10 (50)	
	Do not know	1 (5)		3 (15)	
**Insurance, n (%)**					
	No insurance	1 (5)		2 (10)	
	Private/commercial	15 (79)		15 (75)	
	Medicare/Medicaid	3 (16)		2 (10)	
	Other	0 (0)		1 (5)	

^a^Includes self-employed.

**Table 3:** Patients’ clinical characteristics by phase.

Clinical characteristics		Phase 1 (n=19)	Phase 2 (n=20)
Age at diagnosis (years), median (range)		41.5 (26-49)	44.5 (35-49)
Health literacy, median (range)		4 (1-4)	3.7 (1-4)
Technology literacy, median (range)		4 (1.6-4)	3.7 (2-4)
**Type of cancer, n (%)**			
	Colon	11 (58)	11 (55)
	Rectal	6 (32)	9 (45)
	Do not know	2 (11)	0 (0)
**Cancer stage, n (%)**			
	Stage 0	1 (5)	1 (5)
	Stage 1	0 (0)	1 (5)
	Stage 2	1 (5)	2 (10)
	Stage 3	7 (37)	7 (35)
	Stage 4	7 (37)	9 (45)
	Do not know	3 (16)	0 (0)
**Treatment history, n (%)**			
	No treatment	2 (11)	0 (0)
	Received treatment^a^	17 (89)	20 (100)
Active cancer treatment, n (%)		9 (47)	7 (35)
Ashkenazi Jewish ancestry, n (%)		3 (16)	1 (5)
Family history of early-onset colorectal cancer, n (%)		2 (11)	0 (0)
Awareness of genetic counseling, n (%)		10 (53)	9 (45)
Awareness of genetic testing, n (%)		15 (79)	17 (85)
Awareness of hereditary cancers, n (%)		12 (63)	13 (65)
Awareness of Lynch syndrome, n (%)		4 (21)	7 (35)
**Importance of genetic information for prevention** **, n (%)**			
	Very	18 (95)	15 (75)
	Somewhat	1 (5)	4 (20)
	A little	0 (0)	1 (5)
	Not at all	0 (0)	0 (0)
**Importance of genetics for early cancer detection** **, n (%)**			
	Very	18 (95)	18 (90)
	Somewhat	1 (5)	2 (10)
	A little/not at all	0 (0)	0 (0)
History of genetic counseling, n (%)		8 (42)	7 (35)
History of genetic testing, n (%)		10 (53)	16 (80)

^a^Treatment included surgery, chemotherapy, radiation, and immunotherapy.

**Table 4:** Providers’ demographic and clinical characteristics.

Providers			Phase 1 (n=6)		Phase 2 (n=7)
Age (years), median (range)			39 (31-46)		40 (31-51)
Spanish-preferring, n (%)			1 (17)		0 (0)
**Gender, n (%)**					
	Female	3 (50)		7 (100)	
	Male	3 (50)		0 (0)	
**Race, n (%)**					
	White only	6 (100)		3 (43)	
	Black	0 (0)		1 (14)	
	Asian only	0 (0)		2 (29)	
	Other	0 (0)		1 (14)	
Hispanic ethnicity, n (%)			1 (17)		0 (0)
**Marital status, n (%)**					
	Married/partnered	5 (83)		7 (100)	
	Single	1 (17)		0 (0)	
**Type of provider, n (%)**					
	Physician (MD)	4 (67)		2 (29)	
	Board-certified genetic counselor	2 (33)		2 (29)	
	Physician assistant	0 (0)		2 (29)	
	Nurse practitioner	0 (0)		1 (14)	
Years working with patients with colorectal cancer, median (range)			11 (2-13)		10 (4-17)
**Proportion of time communicating about the genetic risk to patients, n (%)**			6 (100)		5 (71)
	<10%	0 (0)		1 (14)	
	10%-29%	0 (0)		1 (14)	
	30%-49%	0 (0)		0 (0)	
	50%-69%	1 (17)		0 (0)	
	≥70%	5 (83)		5 (71)	
**Proportion of time referring patients to genetic services, n (%)**					
	<10%	0 (0)		2 (29)	
	10%-29%	2 (33)		1 (14)	
	30%-49%	0 (0)		0 (0)	
	50%-69%	3 (50)		1 (14)	
	≥70%	1 (17)		3 (43)	
**Proportion of time seeing patients with** **early-onset colorectal cancer** **, n (%)**					
	<10%	0 (0)		1 (14)	
	10%-29%	3 (50)		4 (57)	
	30%-49%	1 (17)		1 (14)	
	50%-69%	2 (33)		1 (14)	
	≥70%	0 (0)		0 (0)	
**Proportion of time working with racially/ethnically minority patients, n (%)**					
	<10%	0 (0)		0 (0)	
	10%-29%	3 (50)		2 (29)	
	30%-49%	1 (17)		3 (43)	
	50%-69%	2 (33)		2 (29)	
	≥70%	0 (0)		0 (0)	

#### Phase 2

We contacted a total of 51 patients with early-onset CRC and 15 providers, of whom 20 patients and 7 providers completed the survey and interview (Figure 3). The most common reasons for nonparticipation were the same as in phase 1. The median age of phase 2 patients was 47 (range 36-59) years, with about half being female, 4 out of 20 (20%) identifying as Black, 7 (35%) as Hispanic, and 4 (20%) as Spanish-preferring (Tables 2 and 3). Most patients had at least some college education (n=18), were employed (n=13), and had health insurance (n=18). Patients also reported adequate health literacy (median 3.7, range 1-4) and technology literacy (median 3.7, range 2-4). Similar to phase 1, before completing Nest-CRC, 15 (75%) and 18 (90%) patients indicated that GT was very important for cancer prevention and early detection, respectively. However, only 9 (45%) and 17 (85%) reported awareness of GC and GT, respectively.

### Nest-CRC Findings and Recommendations

#### Nest-CRC Quantitative Data

In phase 1, 9 patients reported no history of GT, and after completing the education, 7 (78%) were interested in GT, while 2 (22%) were unsure; none of the participants reported having no interest in GT. In phase 2, 4 patients reported no history of GT, and all of them indicated interest in GT after completing Nest-CRC. Across both phases, most participants reported that each of the Nest-CRC modules was useful and easy to use (phase 1: 23/25, 92%, to 25/25, 100%; phase 2: 24/27, 89%, to 27/27, 100%; Table 5). The average completion time for patients in phase 2 was 11 (range 5-26) minutes.

**Table 5:** Comprehension and usefulness of each Nest-CRC module.

Modules		Phase 1, n (%)		Phase 2, n (%)	
		Patients (n=19)	Providers (n=6)	Patients (n=20)	Providers (n=7)
**Hereditary colorectal cancer, n (%)**					
	Easy to understand	19 (100)	5 (83)	18 (90)	7 (100)
	Helpful	19 (100)	4 (67)	17 (85)	7 (100)
**Genetic testing** **, n (%)**					
	Easy to understand	19 (100)	5 (83)	18 (90)	7 (100)
	Helpful	19 (100)	6 (100)	19 (95)	7 (100)
**Benefits and risks, n (%)**					
	Easy to understand	18 (95)	6 (100)	19 (95)	7 (100)
	Helpful	19 (100)	5 (83)	19 (95)	7 (100)
**Care recommendations, n (%)**					
	Easy to understand	19 (100)	5 (83)	18 (90)	7 (100)
	Helpful	18 (95)	6 (100)	18 (90)	7 (100)
**Family members implications, n (%)**					
	Easy to understand	19 (100)	6 (100)	19 (95)	7 (100)
	Helpful	19 (100)	6 (100)	19 (95)	7 (100)

#### Qualitative Interviews

##### Attraction/Visual Appeal

Participants in both phases reported finding Nest-CRC visually appealing and well-suited to its goals. The layout was described by phase 1 participants as “straightforward,” “concise,” and “clean” (Table 6). Phase 1 participants appreciated how each slide presented information in “bite-sized” amounts, making it “easy to digest and read” and helping to prevent feelings of being overwhelmed, which echoed the quantitative findings. Recommendations for improvement included enhancing the “dark mode” to increase readability and incorporating more visual elements (eg, photos, animations, and diagrams) to maintain attention and simplify complex concepts.

**Table 6:** Interview excerpts by study phase.

Theme	Phase 1	Changes implemented for phase 2	Phase 2
Attraction/Visual Appeal	“I really think it’s concise. I think you’ll lose people if you make them read through too much information even when it’s important, so I thought it was – it was a good capture of the important information.” [Participant #1219, patient] “Maybe having something graphic might make it a little bit better. Because it’s a lot of text. So, I don’t know if maybe having either, like, a little video or animation, at least just for the introduction, that explains what genes are – and mutations or variants – are. That might be helpful.” [Participant #1301, provider]	Included more images and graphics Simplified complex concepts Used colors and font size to highlight important information Improved readability for dark mode	“Yes, I had an 8-year-old child at the time I was making that decision and wanted to know what would impact him the most [...] I thought it was pretty comprehensive [...] I found that very useful, especially having a child that I feel like, anything I needed – I wanted to know everything I could know.” [Participant #2206, patient] “I thought the technology was good, simple, you click, next, go to the next one, and when you finish number 1 it takes you to number 2. My question is, is that going to be the format you are going to use? Is it going to be that shape and color? It's a little bit boring.” [Participant #2304, Spanish-speaking patient]
Comprehension	“Yes, because I guess it said that you could be discriminated against. Obviously, that’s a huge red flag in my opinion. So, that would be the only thing. It didn’t really say much about it. So, that would be the only thing to deter me from getting it because, obviously, I’d have cancer. So, it’s hard to get insurance period. So, if that makes it even harder to get insurance or my children hard to get insurance, then I wouldn’t wanna get it. Or I would need it explained to me a little bit more so that I would know it’s not really that big of a deal or it is a big deal.” [Participant #1202, patient]	Included introduction with summary information about the importance and benefits of GT^a^, what to expect from the education, and the next steps for GT Added more information about sporadic, familial, and hereditary cancer, GT versus somatic/tumor testing, incidental findings from GT, and insurance discrimination Simplified information and provided examples about GT, patients who are at greater risk/in need of testing, and treatment recommendations based on results Included additional optional information about genes, most common types of hereditary colorectal cancer syndromes, GC^b^, and insurance discrimination Added word definitions Explained that cancer-causing gene variants are also known as gene mutations and referred to them as mutations throughout. Added at the end a summary of the information covered	“I thought that the explanations were really easy to understand for people like myself not in the medical field [...] So, it was really easy to understand. And I think kinda gave us a lot of information but not make it overwhelming.” [Participant #2203, patient] “I wanna suggest if there's any data about how minorities are hit pretty hard with colon cancer. If you could possibly put something like that in there because I know sometimes myself – I'm an African American – sometimes minorities feel a little bit afraid about doing the GT [...]” [Participant #2215, patient]
Cultural Acceptability	“We know that this population [cancer patients under 50], they have different needs about treatment and other things and this is something that would be very helpful for this population.” [Participant #1106, provider] “I think the text was short enough that it was easy to read through, but I guess maybe having audio in case people, I don't know, can't read well.” [Participant #1102, provider]	Added voice-over feature	“I think the language is quite basic, and concise, but very appropriate. I don't think some words are difficult to understand for a person from Peru or Venezuelan or Argentinean. The vocabulary is easy to understand. I didn't see any questionable vocabulary.” [Participant #2304, Spanish-Speaking patient] “[A language option] would be easier for [my mother and grandmother]. [...] Maybe having that little option might be better for them and more comfortable for them to participate.” [Participant #2218, patient]
Self-Efficacy	“It is easy to use, easy to navigate, bite-sized bits of information, which is completely different from all the other information you're getting in this process, quick, structured well, and it does take you towards the information you need to make a decision.” [Participant #1213, patient] “I think another reason I have felt more comfortable about it is because my provider had mentioned it to me and kinda talked about it, so I kind of understood it. So, it was just basically like an extra confirmation.” [Participant #1215, patient] “Well, I guess that's one comment then. That wasn't clear to me. [...] I don't remember seeing like if you don't have questions and want to proceed with testing, we can do it today versus seeing a counselor. And like, and then you see a counselor if you have any positives, or you could just see a counselor before testing because that wasn't clear to me. [...]” [Participant #1102, provider]	Added information about GT cost, data security, GT/GC process and wait time, and behavioral and environmental cancer risk factors Added and simplified information about genetic test results and care implications Reordered the content so personal and family benefits will be presented before the risks Insurance discrimination information was simplified	“I mean, honestly overall it’s a thing of – it’s a positive thing overall. It’s just – it’s simple to be done and there’s not – there won’t be repercussions for finding out information [...] ‘Cause that’s what something that people worry about is – so, if I find out this is a hereditary thing are my kids gonna be denied whatever, insurance or whatever down the road because I did this now [...] So, you know – I think between that and how simple the test – knowing that there won’t be those repercussions and knowing how simple the testing is I think that was a good message for me.” [Participant #2208, patient] “And that’s the tricky part. If somebody gets diagnosed with colon cancer, the first they are thinking is am I going to live? Am I going to not live? And what’s the treatment? They’re not truly thinking about genetics. So, maybe once they met with the surgeon and oncologist and have had a treatment plan. At that time maybe a good way to bring in that. We have universal protocol anyways for colon cancer. Every colon cancer should be tested. So, it has to be brought up at some point. [...]” [Participant #2101, provider]
Usability/Utility	“I think the app is pretty simple to navigate... I think overall, this does a pretty good job of making sure that somebody at 50 that doesn’t have a whole lot of experience with technology and somebody at 20 who basically that’s all he does is technology are able to use it. Again, I think it’s pretty good across the board, but you’re still gonna have some outliers.” [Participant #1210, patient] “Yes, because it was difficult for me because I needed my daughter’s help and when my daughter wasn’t there, I couldn’t enter because I would copy the information and I would go to a link, and it wouldn’t let me enter. Yes, it was difficult to me... Oh, that [being sent a text with a direct link] would be easier for those of us who don’t know that much about technology. It would be easier where there is just a link, and you click on it and it takes you to the information.” [Participant #1306, Spanish-speaking patient]	The education was sent via SMS text message, and a direct link was provided via email or SMS text message An audio option was implemented Added an option to obtain additional information	“I did like that it allows for audio and visual learning. That was kinda one of my favorite parts because a lot of times, essentially, some patients can hear what you have to say, but the ability to be able to kinda pause and listen or go back and listen again, I think that was a very smart use of the learning modality. I thought that the information was very clear and concise, and it wasn't very cumbersome. It wasn't just a lot of information on each slide.” [Participant #2105, provider] “A circle that came out there? I could see it moving but I didn't know what it was to read it for me. I thought they were listening to me. I kept reading and doing the up and down but I didn't use it to listen.” [Participant #2301, Spanish-speaking patient] “But I guess maybe what might be helpful is – I don’t know how to say this. Sorta like an outline or something. You know what I mean? So that you can skip forward or skip back [...].” [Participant #2203, patient]

^a^GT: genetic testing.

^b^GC: genetic counseling.

The main factors most patients and providers emphasized in phase 2 were a desire to protect family members and trust in science and medical providers. Patients stated that a recommendation from a provider to review the education would influence their interest in it. The information provided in the education, along with its ease of use, was highlighted in phase 2 as the primary factor that attracted participants.

For the most part, participants did not have strong opinions about the visuals of the tool. Most described it as “clean” and “easy to navigate” and reported that the text was clear and easy to read. One participant found the aesthetic dull and “boring” and felt it needed more color. Another participant mentioned that the lack of personal interaction decreased their interest, preferring to speak with a person instead.

##### Comprehension

Participants in both phases found the information easy to comprehend and described the intervention as “straightforward,” “simple,” “succinct,” “understandable for a layperson,” and “super easy to understand.”

Nearly all phase 1 patients stated that they understood GT could be used to determine genetic predisposition for their CRC and that learning whether they carry a particular gene could be helpful for family members. The most commonly cited point of confusion in phase 1 was the possibility of discrimination based on their GT results. This was surprising to many participants, with some requesting links or resources for further information. Other recommendations from phase 1 participants included adding information on the most common types of hereditary CRC syndromes (eg, Lynch syndrome, familial adenomatous polyposis), clarifying which patients are most at risk or in need of testing, distinguishing between GT and somatic/tumor testing, addressing incidental findings from GT (eg, beyond CRC, such as BRCA1), defining specific gene associations, discussing treatment options based on variants, reducing redundancies in “informed consent,” and communicating clearly to lay audiences (eg, clarifying language and providing examples). When suggested by the interviewer, patients agreed that a dictionary tool to define unfamiliar terms would be useful.

Phase 2 participants felt the intervention provided just the right amount of information: educational, yet not overwhelming for a new patient. Most phase 2 providers found the intervention easy to understand, free of jargon, and containing an appropriate level of detail.

Although participants were overall very satisfied, they suggested some additional educational topics. One of the most commonly requested topics by both patients and providers was information on what other cancer and health condition risks can be detected via GT. The general consensus was that most patients would want GT to detect different cancers if it were covered by insurance or affordable. Some participants raised privacy concerns regarding who can access their results and what can be done with their DNA and results in the future. Many providers suggested that it could be beneficial to outline privacy policies within the intervention. Participants were also interested in the impact and next steps for family members based on their GT results. One participant requested more data on cancer prevalence by race/ethnicity.

Some participants wanted more information (eg, the likelihood of having Lynch syndrome, more details about specific genes, and variants of uncertain significance), while others felt the level of information provided was sufficient and that additional details should be discussed with a genetic counselor. In both phases 1 and 2, there was disagreement regarding the preferred term “variant” versus “mutation” and when to use each.

##### Cultural Acceptability

All participants in phase 1 and phase 2 indicated that they felt the information was acceptable to a wide audience of varied backgrounds. None reported concerns that the content of Nest-CRC was offensive or inappropriate for any groups. Instead, participants described the material as “neutral,” “broad,” and believed “it can help anyone.”

Most of the concerns regarding the acceptability of Nest-CRC in phase 1 were related to accessibility. English-preferring participants inquired whether the education was available in Spanish and emphasized the need for patient-friendly language. Participants found the online delivery to be appropriate, given that the target audience is under 50 years of age, but recommended reevaluation if Nest-CRC is expanded to older patients. Participants also suggested adding an audio voice-over option or videos within Nest-CRC to improve accessibility for blind people/those with visual impairment, those not fully literate in English, or those who prefer listening over reading.

One phase 2 participant appreciated that the materials were inclusive of adopted patients who may be unaware of their family history. Phase 2 participants were also appreciative of the audio voice-over option.

##### Self-Efficacy

Both phase 1 and phase 2 participants described Nest-CRC as “very informative” and “comprehensive,” and most felt that the intervention provided enough information to help them decide if they wanted GT without being overwhelming. Most patients had already undergone GT or were interested in it, evaluating Nest-CRC as a helpful way to be “proactive,” with some wishing it had been available when they were first diagnosed.

Trust in their care team’s recommendations was a major facilitator for using Nest-CRC. Participants responded positively when asked if they would complete the intervention if their provider recommended it, including patients who were undecided about GT. A few participants did not fully understand the benefits of GT (eg, therapeutic decision-making, risk management recommendations, and cascade testing), given that they already had cancer, with one commenting that it would be “pointless” now. Other potential barriers to GT included cost, information sharing/data security, and fear and anxiety around test results. Some participants also described fear of insurance discrimination as a potential barrier and felt this risk should be better clarified.

Self-efficacy for phase 2 participants was bolstered by their greater satisfaction with the information provided about insurance, costs, and the risks of discrimination, as well as the available legal protections. Participants identified information on screenings and sample collection modalities (saliva vs blood) as important topics to support GT decision-making. Many providers and patients noted an assumption that GT was more invasive or complicated than it is and felt that providing information about the minimally invasive process could increase interest. The option for saliva testing was highlighted as important to make GT more appealing to patients afraid of blood and needles and more manageable for those undergoing multiple treatments or procedures for cancer care.

For phase 2, the remaining barriers to GT decision-making included unresolved concerns about discrimination and the ability to afford GT. Despite being informed that most insurance plans cover GT, participants remained concerned that their results could be positive and that cascade testing could be recommended for uninsured family members. One patient, who had previously experienced mishandling and misuse of medical results, expressed distrust in the efficacy of policies and institutions despite education about protections. Some newly diagnosed younger patients stated that being diagnosed with cancer was overwhelming and found it difficult to process information and make decisions, even with the necessary resources. With this in mind, some participants recommended that providers bring up or offer Nest-CRC again during the second or third visit to allow patients time to check insurance coverage for GC and GT.

##### Usability/Utility

Participants in both phase 1 and phase 2 described using the Nest-CRC tool as “user-friendly,” “very easy to use,” and “seamless.” They found the tool logically structured and easy to navigate, and appreciated that it is accessible on both desktop and mobile devices.

Phase 1 participants encountered minor technical issues, such as difficulty getting the link to work and not being able to return to where they left off. Participants felt usability could be improved by providing a direct link (rather than requiring copying and pasting into a browser), adding an audio option, allowing easier navigation of content out of sequence, and offering access to Nest-CRC via tablet in the clinic waiting room.

Phase 2 participants who noticed the audio option rated it positively, even if they preferred to read. However, this feature was not apparent to all users. For users with their phone set to “silent,” the audio option was also silenced, even if the phone volume was turned up. This led to confusion among many participants who were unable to hear the audio. One patient even thought the tool was recording audio because they could not hear it and noticed the sound wave animation within the audio button.

#### Summary and Next Steps

Most participants found each of the modules easy to understand and helpful. In general, patients described the content as straightforward, easy to comprehend, beneficial to anyone, informative, and useful for making decisions about GT. Following phase 1, usability was improved by providing participants with a direct link to Nest-CRC, sending links via SMS text message, and adding an audio option. The educational modules were also reordered to highlight the benefits first and end with the risks. Several additions were made to the educational content, including optional pathways and links for more detail on specific topics (eg, Lynch syndrome), definitions of key terms (eg, genes), graphs, images, and clarifying information about GT cost, insurance discrimination, and the GT process.

For the next phase, the audio voice-over will be improved, and additional optional pathways (eg, genes commonly tested in MGPT) will be incorporated. The revision will include more information about variants of uncertain significance, as well as content explaining the difference between GT and somatic testing, and addressing GT privacy concerns. Patients will also have the option to select their preferred language. Navigation within Nest-CRC will be simplified to allow easier return to core educational content from optional paths and modules.

## Discussion

### Principal Findings

Nest-CRC was developed as a user-friendly, scalable digital health tool designed to improve access to GT by facilitating pretest education at a lower cost. Findings indicated strong attraction, comprehension, cultural acceptability, self-efficacy, and usability of Nest-CRC in both phases. Endorsement of Nest-CRC was high, with participants recommending it be offered routinely and repeatedly to patients at different stages of the cancer journey, as some patients may prioritize GT immediately, while others may postpone it until survivorship care. Nest-CRC was also described as an acceptable alternative for empowering patients with information about GT and supporting their decision-making. Therefore, it was identified as a viable strategy for streamlining patients with early-onset CRC toward GT.

### Comparison With Prior Work

NCCN guidelines recommend MGPT for all patients with early-onset CRC [10], but referrals to genetic services for these patients are inconsistent [11,12]. A prior study from 2 Texas health systems, evaluating data from 2009 to 2017, revealed that 58% of patients with early-onset CRC were referred for GC, and only 37% completed GT [11]. A more recent study, examining retrospective data from 2010 to 2019 at Cleveland Clinic, found that 62% of patients with early-onset CRC were referred to GC, 49% completed GC, and 48% completed GT [12]. In our study, less than half of the patients reported a history of GC, while two-thirds reported a history of GT. The proportion of patients reporting GT in our study was higher than previously reported rates for patients with early-onset CRC. However, interviews revealed that some patients were confusing their experiences with somatic testing and germline testing, which may have contributed to the elevated reporting of GT. Among those patients with early-onset CRC who denied GT at baseline, most expressed interest in GT after completing Nest-CRC. Additionally, the few patients who were unsure about GT showed interest in GC. This suggests that Nest-CRC can be used to streamline the triage of patients with early-onset CRC for GT, while complex patients needing more support can be prioritized to maximize the efficiency of limited GC resources.

Prior studies examining barriers to genetic services among patients with early-onset CRC have identified cost, a shortage of qualified genetic professionals, and racial/ethnic referral disparities [11,19]. In our study, patients expressed concerns about the cost of GT, insurance coverage and discrimination, and the potential misuse of their DNA. While the cost of GT remains a concern, it has decreased substantially, making it more affordable for many individuals without health insurance. In the United States, patients who meet insurance criteria for GT typically pay between US $0 and US $100 out of pocket, while those without insurance may pay around US $300. Additionally, some laboratories offer financial assistance. Furthermore, the United States has federal and state laws that protect patients from insurance discrimination. For example, the Genetic Information Nondiscrimination Act (GINA) prevents nonmilitary employers and health insurance companies from using GT results against patients [39]. However, GINA does not apply to life, disability, or long-term insurance companies. Some states, like Florida, have enacted laws that protect patients from life, disability, or long-term insurance companies using GT results against individuals residing in the state [40,41]. Therefore, patients’ commonly reported concerns about GT could be alleviated through education about existing resources and federal and state protections.

To address barriers to accessing GT information and services, the Clinical Genome Resource’s Consent and Disclosure Recommendations working group recommended a brief pretest genetic education approach for more straightforward cases, reserving traditional GC for patients with greater clinical and genetic complexities or those without well-established testing recommendations [20]. Automated educational tools, such as videos and written materials, have proven effective in delivering genetic education, particularly for patients with high-risk cancer [21,22,25,42]. For example, a study involving patients with pancreatic cancer found that when oncology providers used an educational video to obtain GT informed consent, the rate of GT increased 6.5 times compared with previous years, when traditional GC referrals were used [21]. However, these advances still rely on clinic staff and providers to obtain informed consent and order GT.

Nest-CRC provides an alternative for GT education that can further alleviate the burden on patients, clinic staff, and institutions. Like other patient-driven digital tools [42,43], Nest-CRC can be completed conveniently from home, enhancing accessibility without incurring out-of-pocket costs for the patient. For institutions, the annual cost of using Nest-CRC can be tailored to the specific functionalities required, averaging a few thousand dollars per month—less than the cost of an entry-level GC assistant. Unlike a single assistant, Nest-CRC can scale the volume and capabilities of genetic services without being impacted by patient load, staff turnover, or the need for ongoing training. By automating workflows for education, consent, test ordering, and results return, tools such as Nest-CRC can significantly enhance clinic efficiency. While this study focuses on the perceived benefits of pretest genetic education, implementing a platform such as Nest has the potential to generate substantial cost savings and operational efficiencies across other routine tasks, such as family history collection, patient tracking, risk model calculations, and care plan management. As the demand for genetic services continues to grow, digital health tools such as Nest-CRC could be leveraged to identify high-risk patients and promote GT across various health care settings, such as primary care, oncology clinics, and even the general public. In this way, Nest-CRC offers an acceptable alternative strategy to expand equitable access to GT among high-risk patients.

### Strengths and Limitations

The primary goal of this study was to evaluate the reactions and recommendations of English- and Spanish-speaking patients and providers to Nest-CRC, with preliminary data on patients’ interest in GT as a secondary aim. This study is innovative in providing valuable insights from a diverse group of patients and providers, highlighting key considerations for developing pretest genetic education for patients with early-onset CRC. However, due to the small sample size, quantitative data were limited to descriptive purposes and were used primarily to describe the study sample and support the qualitative findings by incorporating patients’ immediate feedback on the educational content. While we intentionally recruited Black and Spanish-speaking patients to ensure diverse representation, the findings should not be generalized to all patients with early-onset CRC. It is also important to note that during interviews, some participants expressed uncertainty about their GT history; therefore, the self-reported GT data should be interpreted with caution. This uncertainty underscores the potential benefit of a comprehensive digital health platform where GT history and related lifelong care recommendations can be easily accessed and shared with patients’ clinicians and family members as needed.

### Conclusions and Future Directions

Adults with early-onset CRC are at higher risk for having a hereditary cancer syndrome. GT to identify the causative variant can facilitate screening and risk reduction measures for both patients and their relatives. Despite GT being recommended for all patients with early-onset CRC, racial disparities persist in referrals for GT, access to GC, and uptake of both GC and GT. These issues are further compounded by a shortage of qualified genetic professionals and patients’ concerns about the cost of genetic services. Our findings suggest that Nest-CRC is a promising strategy to scale genetic services by augmenting pretest genetic education and promoting GT uptake among patients with early-onset CRC from diverse racial and ethnic backgrounds. Future studies should implement digital GT platforms in clinical settings to evaluate their feasibility and acceptability among high-risk patients and their relatives from diverse racial and ethnic backgrounds, as well as assess their impact on lifelong care recommendations and survival outcomes.

## Abbreviations

ASCO: American Society of Clinical Oncology
CRC: colorectal cancer
GC: genetic counseling
GINA: Genetic Information Nondiscrimination Act
GT: genetic testing
HINTS: Health Information National Trends Survey
MGPT: multigene panel testing
NCCN: National Comprehensive Cancer Network

## Data Availability

Deidentified data generated and analyzed during this study will be made available (as allowable according to institutional review board standards) by emailing the corresponding author.
